# Hydroxypropyl Cellulose Hydrogel Containing *Origanum vulgare* ssp. *hirtum* Essential-Oil-Loaded Polymeric Micelles for Enhanced Treatment of Melanoma

**DOI:** 10.3390/gels10100627

**Published:** 2024-09-29

**Authors:** Katya Kamenova, Ivan Iliev, Anna Prancheva, Pencho Tuleshkov, Krasimir Rusanov, Ivan Atanassov, Petar D. Petrov

**Affiliations:** 1 Institute of Polymers, Bulgarian Academy of Sciences, 1113 Sofia, Bulgaria; kkamenova@polymer.bas.bg (K.K.); a_prancheva@polymer.bas.bg (A.P.); pen.tul@polymer.bas.bg (P.T.); 2 Institute of Experimental Morphology, Pathology and Anthropology with Museum, Bulgarian Academy of Sciences, 1113 Sofia, Bulgaria; taparsky@abv.bg; 3 Department of Agrobiotechnology, AgroBioInstitute, Agricultural Academy, 1164 Sofia, Bulgaria; krusanov@abv.bg (K.R.); ivan_atanassov@abv.bg (I.A.); 4 Centre of Competence “Sustainable Utilization of Bio-Resources and Waste of Medicinal and Aromatic Plants for Innovative Bioactive Products” (CoC BioResources), 1000 Sofia, Bulgaria

**Keywords:** oregano essential oil, polymeric micelles, nanocomposite hydrogel, hydroxypropyl cellulose, skin cancer

## Abstract

*Origanum vulgare* ssp. *hirtum* essential oil (OEO) is a natural oil with high therapeutic potential. For some applications, however, the development of novel formulations is still needed to improve the bioavailability and stability of OEO. In this study, we describe the fabrication of an original nanocomposite hydroxypropyl cellulose (HPC) physical hydrogel, containing OEO-loaded polymeric micelles, for topical delivery. The concentration of the main active compounds of OEO—carvacol and thymol—was determined using gas chromatography (GC) analysis. OEO was first encapsulated into Pluronic F127 micelles, and then embedded into HPC gel. Micellar and gel formulations of pure polymers and OEO-containing systems were characterized by dynamic light scattering (DLS) and rheology measurements, respectively. Selected formulations were evaluated for cytotoxicity and antiproliferative activity. The hydrogel formulation of HPC with micellar OEO (8% HPC, 2% F127, 1% OEO) exhibited sustained release of the oil and selectivity towards SH-4 tumor cells (an in vitro model of melanoma).

## 1. Introduction

Skin cancer is one of the most common cancers, the incidence of which has dramatically increased in recent years worldwide. There are two groups of skin cancer—non-melanoma (NMSC) and melanoma (malignant melanoma; MM). Although MM is relatively rare (around 1.7% of skin cancers) compared to NMSC, it is the most aggressive type of skin cancer, causing more than 75% of all skin cancer deaths [1,2,3]. Earlier diagnosis of this type of cancer could lead to better results in its treatment and a reduction in mortality. Currently, the therapy of cutaneous melanoma includes various conventional methods such as tumor resection, radiation therapy, local therapy and chemotherapy, as well as targeted therapy and immunotherapy. However, not all patients respond to these therapies, and in most cases the treatment is ineffective. Among the reasons indicated by the scientists and doctors as limiting factors for efficient therapy are drug resistance, lack of selectivity, systemic toxicity and unwanted side effects. Therefore, continuous efforts are needed for the search for, and improvement and development of, new and effective means for the treatment of this disease [4,5,6,7,8]. Nanotechnology, and particularly the development of drug nanocarriers, is encouraging for the delivery of drugs with improved targeting, reduced toxicity and increased permeability and retention [9]. On the other hand, topical chemotherapy, compared to oral and parenteral chemotherapy, has some advantages in terms of reducing the dose-dependent systemic side effects and toxicity of chemotherapeutic agents [10].

In recent years, the use of natural compounds as antitumor agents has become an area of great interest [11]. Plant extracts and their active compounds have been used for their medicinal properties since ancient times, and nowadays attract more attention [12]. Essential oils (EOs) are natural aromatic liquids obtained as secondary metabolites of aromatic plants, being a complex mixture of volatile lipophilic compounds with low molecular weight. The variety of chemical constituents found in EOs (terpenoids, flavonoids, aldehydes, phenols, carotenoids and alkaloids, etc.), provide a wide spectrum of activities—antioxidant, anti-inflammatory, antifungal, cytoprotective, antitumor, antidepressant, antimicrobial and antiviral. Thus, the therapeutic potential of EOs has been widely studied in various pathologies. They have found numerous applications in the perfume, cosmetic, food and pharmaceutical industries as well as in medicine [13,14,15,16,17,18]. Studies carried out on in vitro and in vivo models have demonstrated that EOs possess the ability to control the proliferation of cancer cells, which makes them promising agents for topical use in treatment of melanoma and non-melanoma skin cancer [19]. However, these phytochemicals have a number of disadvantages, such as high volatility, limited stability, poor solubility in water, high risk for allergic reactions and respiratory disorders and risk of toxicity, which limits their wide use in therapies [16,20,21,22]. The encapsulation of EOs into polymer-based formulations (micelles, micro- and nanocapsules, films or hydrogels) can enhance their stability, solubility and bioavailability. Furthermore, the use of nanoformulations can significantly increase the therapeutic effectiveness and safety profile of EOs because controlled release at a target zone can be achieved [16,23,24,25,26]. In addition, nanocarriers can solubilize the hydrophobic bioactive substances, and deliver them to the tumor cells by passive (enhanced permeation and retention (ERP) effect) or active (specific ligands functionalized nanoparticles) targeting mechanisms [25,27,28]. For instance, the use of polymeric micelles for dermal drug delivery has demonstrated some positive effects including increased bioavailability, improved drug efficacy, delivery of drugs to specific cells and sustained release over an extended period [29]. Polymeric micelles from the commercially available copolymers Poloxamers (Pluronics) are widely studied as skin drug-delivery systems [30,31,32,33]. Poloxamers are biocompatible, non-toxic synthetic amphiphilic triblock copolymers approved by FDA for medical use. Usually, these copolymers consist of one middle thermosensitive block from poly(propylene oxide) and two outer blocks of poly(ethylene oxide). Thus, at room temperature, Poloxamers can self-assemble in aqueous media and the formed “core-shell” micelles solubilize the hydrophobic bioactive components of EOs [34,35,36]. However, the use of a liquid formulation of Poloxamer micelles for topical treatment of skin cancer may result in compromised stability and limited retention time. In this aspect, hydrogel-based formulations are preferred for local skin cancer treatment as they can provide free-standing systems and sustained drug release. In addition, combinatorial formulations of nanocarrier-loaded gels (nanocomposite gels) are of special interest because they allow for a combination of different natural and/or synthetic constituents, which makes it possible to modulate and improve the characteristics of the system [25]. Indeed, polysaccharide hydrogels have gained interest in biomedicine as drug delivery systems because they are a convenient platform for embedding different nanocarriers pre-loaded with active molecules [37]. Hydroxypropyl cellulose and its derivatives are particularly important for pharmaceutical applications because they are biocompatible, biodegradable, bioadhesive and form reversible physical hydrogels with extraordinary characteristics, such as a high content of water and good texture, spreadability and skin flexibility [38]. In our previous work, we reported the successful elaboration of a novel in situ gelling hydroxypropyl cellulose formulation comprising cannabidiol-loaded block copolymer micelles, which exhibited pronounced activity against malignant MCF-7 human tumor cells [39].

Oregano essential oil (OEO) is extracted from Origanum species by steam distillation [40]. A large part of OEO production is related to *O. vulgare* ssp. *hirtum* (known as Greek oregano) due to its high EO concentration and quality [41,42]. The composition of OEO includes various compounds—acyclic, monocyclic and bicyclic monoterpenes, sesquiterpenes, etc. It should be mentioned that the concentration of each component depends on various factors such as species, geographical location, climate and growing conditions, pests, soil conditions and harvest time. Nevertheless, the main constituents of OEO are the monoterpenes carvacrol (2-methyl-5-(1-methylethyl)-phenol) and thymol (2-isopropyl-5-methylphenol), and the therapeutic properties of the oil are commonly attributed to these two compounds. OEO is well-known for its antibacterial, antifungal and antioxidant properties, as well as its anticancer and anti-inflammatory activities [26,41,42,43,44,45]. It is used for stomach-ache, respiratory disorders, painful menstruation, rheumatoid arthritis, etc. [42]. The inhibitory effects of OEO against different pathogenic bacteria, both Gram-positive and Gram-negative strains, have been widely studied. The essential oil has been reported to be effective against *Staphylococcus aureus*, *Staphylococcus epidermidis*, *Streptococcus pyogenes*, *Streptococcus mutans*, *Streptococcus pneumoniae*, *Bacillus cereus*, *Enterococcus faecalis*, *Listeria monocytogenes*, *Propionibacterium acnes*, *Escherichia coli*, *Pseudomonas aeruginosa*, *Klebsiella pneumoniae*, *Helicobacter pylori* and *Salmonella enteritidis* [46,47,48]. Additionally, research has shown the powerful antifungal effects of OEO on Candida albicans, Trichophyton rubrum, Trichophyton mentagrophytes, Aspergillus flavus, Malassezia furfur, Penicillium funiculosum and Penicillium ochrochloron [49,50,51]. Some works have focused on other biological activities of OEO [26,43]. An in vitro study by Cardile et al. confirmed the antioxidant and anti-inflammatory activity of OEO as well as its tissue remodeling and wound healing capacity on human keratinocytes NCTC 2544. The oil acted as a potent inhibitor against pro-inflammatory damage from IFN-γ and histamine in human keratinocytes and showed improved cell motility during tissue healing [52]. The anticancer activity of OEO has been reported against the proliferation of several types of cancer cells, including breast adenocarcinoma (MCF-7), cervical adenocarcinoma (HeLa), colon adenocarcinoma (HT-29) and urinary bladder carcinoma (T24) [53,54,55,56,57]. Nanni et al. investigated the antineoplastic effect of an *O. vulgare* L. ssp. (from Mount Athos (Greece)) *hirtum* phytocomplex against murine (B16–F10) and human (A375) melanoma cells. The results showed that an *O. vulgare* hydroalcoholic extract inhibits melanogenesis and melanoma cell proliferation, triggering programmed cell death (apoptosis and necroptosis) in A375 human melanoma cells via mitochondria and DNA damage [8]. However, the biological activity of OEO can be compromised due to its high volatility and poor stability and solubility. Hence, novel strategies are still needed to improve the characteristics of OEO-based formulations for biomedical applications [58]. Taking the advantages of nanotechnologies, we developed a novel HPC-based nanocomposite hydrogel formulation of oregano essential oil for local treatment of melanoma. OEO was first loaded into core–shell polymeric micelles and the colloid particles were then embedded into the HPC hydrogel matrix. The cytotoxicity, antiproliferative activities and selectivity of formulated and pure OEO against SH-4 tumor cells (an in vitro model of melanoma) were studied in detail. As far as we know, this is the first work reporting a higher selectivity of the formulated OEO against human melanoma cells as compared to the pure oil.

## 2. Results and Discussion

**Figure 1:**
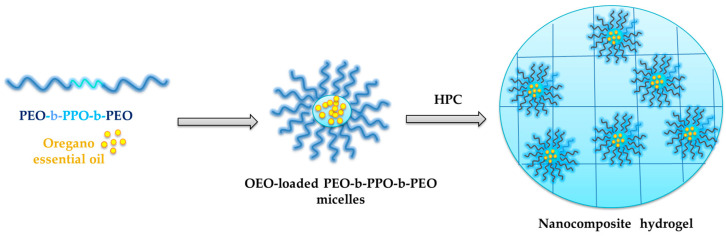
Schematic representation of the preparation of nanocomposite hydrogel containing *Origanum vulgare* ssp. *hirtum* essential-oil-loaded polymeric micelles (HPC-Plu-NP-OEO).

The nanocomposite hydrogel for topical delivery of *Origanum vulgare* ssp. *hirtum* essential oil was prepared by a facile procedure as summarized in Figure 1. OEO was first encapsulated by polymeric micelles through hydrophobic interactions with the cores of poly(ethylene oxide)-b-poly(propylene oxide)-b-poly(ethylene oxide) (Pluronic F127) copolymer micellar nanocarriers, and then incorporated into a physical hydrogel of hydroxypropyl cellulose.

### 2.1. Characterization of Origanum vulgare ssp. hirtum Essential Oil

The chemical composition of OEO was determined by GC–MS analysis (Supplementary Information, Table S1). The most important compounds—carvacrol and thimol, responsible for the antioxidant, antibacterial, antifungal and anticancer activities of OEO [43,44,45]—were quantified before and after loading into polymeric micelles. Data from GC–MS analysis is present in Table 1, expressed as % of the total peak areas. This test confirmed that the procedure used for the loading of OEO into micellar carriers is gentle and did not alter the composition of the oil.

### 2.2. Preparation and Characterization of OEO-Loaded Polymeric Micelles

The successful preparation of OEO-loaded polymeric nanocarriers was achieved by the solvent evaporation method. Pluronic F127 and OEO were dissolved in ethanol and the solution was added to deionized water. After that, the organic phase was evaporated. In aqueous media, the lipophilic OEO molecules interact with the core-forming PPO segments of copolymer via hydrophobic interactions. As a result, OEO was embedded in the cores of F127 micelles, which increases its solubility in water and can protect the bioactive compounds of the oil from degradation [59]. To assess the loading capacity of the carriers, six samples with different mass ratios F127/OEO (2, 1.8; 1.7; 1.5; 1.2 and 1) were prepared, at a constant concentration of F127 = 1% *w*/*w*. At first glance, the turbidity of the system was dramatically affected by the composition (Table 2, Figure S1). Stable colloids were formed when the content of OEO was relatively low (mass ratio 2, 1.8; 1.7). DLS analysis of these samples revealed nano-sized particles with slightly negative ζ potential. The decrease in the polymer/oil mass ratio from 2 to 1.7 resulted in a notable enlargement of the hydrodynamic diameter (D_h_) of the nanoparticles from 25 nm to 60 nm and a decrease in their surface charge (Table 2). The size distribution plots of empty (Plu-NPs) and OEO-loaded micelles (Plu-NP-OEO) are shown in the Supplementary Information (Figure S2). Generally, the dynamic aggregates of F127 tend to undergo structural rearrangement aiming to encapsulate the OEO and to minimize the free energy of the system by avoiding water–oil interaction. When the loading capacity of nanocarriers is overreached, the OEO fraction, which cannot be encapsulated by the micelles, tends to form small oil droplets. The system loses its equilibrium state and after a certain period of time a phase separation occurs. In our case, the decrease in the F127/OEO mass ratio to 1.5 yielded a very turbid suspension, which was unstable upon storage for several days (Table 2; Figure S1). Among the samples studied, the colloid with a double excess of F127 (sample 1) exhibited characteristics (small particle size and negative surface potential) which are favorable for further incorporation in hydrogels.

Next, a micellar form of OEO, containing two times more oil (compared to sample 1 in Table 2), was prepared by keeping the same polymer/OEO mass ratio of 2. The sample had a monomodal size distribution with D_h_ = 32 nm and ζ potential −4.80 mV. It was stable for more than 2 months. The successful preparation of a more concentrated OEO micellar form makes possible the incorporation of high portions of the active substance into the hydrogel carrier.

### 2.3. Preparation and Characterization of HPC Hydrogels, HPC-Plu-Nanocomposite Hydrogels and OEO-Loaded HPC-Plu-Nanocomposite Hydrogels

Initially, aqueous solutions/gels of pure HPC (1–10% *w*/*w*) were prepared by dissolving HPC into deionized water and vigorously stirring for 20 min at room temperature. After that, the samples were kept overnight at 25 °C before measurements. Key parameters of hydrogels used in topical applications are the deformation and flow pattern, as they provide an idea for the ability of the gel formulation to spread on the skin [25]. The properties of all prepared aqueous HPC solutions/gels were examined by dynamic rheology measurements. The measurements were performed at a temperature of 32 °C, which simulates wound conditions. At low HPC concentrations (from 1% to 3% *w*/*w*), the samples were in the form of a highly viscous solution and their elastic modulus was lower than the loss modulus (G″ > G′). This means that at these concentrations the viscous response is higher than the elastic response and such carriers are not suitable for application to wounds because they will leak. At higher concentrations of HPC (4% to 10% *w*/*w*), the samples were in the form of a gel as the elastic modulus was higher than the loss modulus (G′ > G″) (Table 3). The complex viscosity of the HPC system increased notably with the increase in polymer concentration from 1 to 10% *w*/*w*.

**Figure 2:**
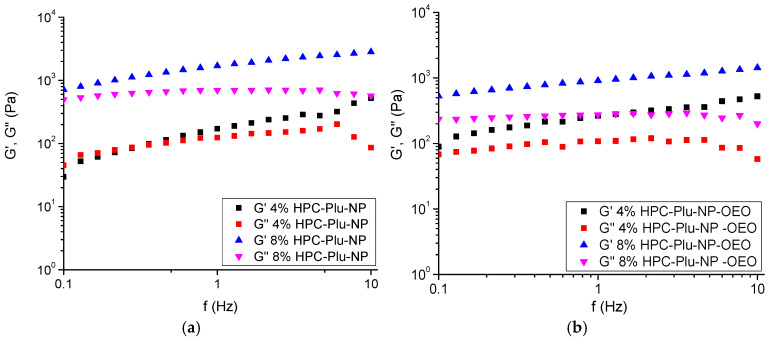
The storage (G′) and loss (G″) moduli of (**a**) Plu-NP-containing nanocomposite hydrogel formulations (8% and 4% HPC-Plu-NP) and (**b**) Plu-NP-OEO-containing nanocomposite hydrogel (4% and 8% HPC-Plu-NP-OEO), measured at 32 °C and constant strain (γ = 0.01) in the frequency range 0.1–10 Hz.

Based on these preliminary tests by dynamic rheological analysis of pure HPC hydrogels, two concentrations of HPC—4% and 8%—were selected for the elaboration of nanocomposite hydrogels and OEO-loaded nanocomposite hydrogels. The nanocomposite systems were prepared by dissolving HPC in micellar solutions, keeping the HPC/F127 mass ratio equal to 4 (8% HPC, 2% Plu-NP, 1% OEO; and 4% HPC, 1% Plu-NP, 0.5% OEO). The prepared solutions were stirred for 30 min at room temperature and then kept overnight at room temperature before measurements. The fabricated HPC-Plu-NP hydrogels were translucent, while OEO-loaded HPC-Plu-NP-OEO nanocomposite formulations were white. All samples exhibited a smooth texture, and phase separation was not detected. Dynamic rheological characterization of nanocomposite hydrogel systems was performed by varying the oscillatory frequencies from 0.01 to 10 Hz, at a constant strain amplitude of 0.01. As show in Figure 2, the hydrogel obtained with 4% HPC has rather close values of the elastic and loss moduli, but it still behaved as a gel-like material (G′ > G″). The hydrogel obtained with 8% HPC possessed much higher G′ than the less concentrated sample (4%), and the values of elastic modulus significantly exceeded those of loss modulus. The presence of polymeric micelles in the HPC network decreased the storage modulus (1741 Pa at 1 Hz) as compared to the pure HPC hydrogels (2341 Pa at 1 Hz) at the same concentration (8%) of gelling polymer. Generally, HPC forms a physical hydrogel above certain critical concentration and temperature via entanglements of macromolecules (junction pints) and hydrogen bonding. It seems that the presence of soft polymeric micelles interfered to some extent with the formation of a strong gel. The effect of OEO-loaded polymeric micelles on the weakening of the polymer hydrogel was even more pronounced. This means that the incorporated soft polymeric particles (with and without oil) occupy a limited space of material and thus hinder the physical interaction of HPC molecules with each other. Nevertheless, OEO-loaded nanocomposite hydrogels were characterized with good elastic behavior (G′ >> G″), and the mechanical strength of the formulations prepared with 8% HPC was much higher than the gel wit 4% HPC.

**Figure 3:**
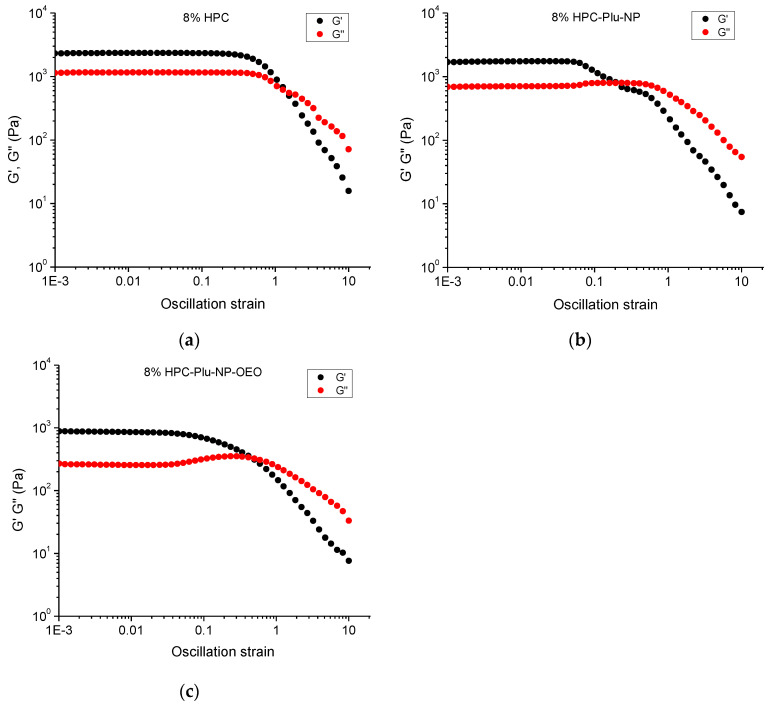
Variation of storage (G′) and loss (G″) moduli at a frequency of 1 Hz as a function of the strain amplitude for: (**a**) HPC-hydrogel (8% HPC), (**b**) micelle-containing nanocomposite hydrogel (8% HPC-Plu-NP) and (**c**) OEO/micelle-containing nanocomposite hydrogel (8% HPC-Plu-NP-OEO).

To assess the ability of the elaborated gel formulations to spread upon applied force, the rheological properties of the selected samples (8%) were studied as a function of the strain amplitude (Figure 3). As is typical for a physical gel, the pure HPC hydrogel exhibited a linear viscoelastic region (0.001 ≤ γ ≤ 0.5) with constant values of the elastic and loss moduli, where G′ > G″. This result indicated that up to a shear strain of 0.5 (at a fixed frequency of 1 Hz), the elastic component dominates over the viscous component and the three-dimensional network structure of the material is preserved. A further increase in the strain amplitude resulted in a gradual decrease in G′ and G″ (Figure 3a), which means that the microstructure of the gel is damaged; some macroscopic cracks are formed and, at certain point, the viscous component becomes dominant (G″ > G′). In other words, when the stress applied to the gel formulation is high enough, the material cannot withstand the applied force and begins to flow [60]. The incorporation of blank and OEO-loaded F127 micelles reduced by one order of magnitude the linear viscoelastic region (Figure 3b,c). In addition, the slight rise of G″ (0.04 ≤ γ ≤ 0.4) indicates a breakdown of Pluronic micellar structures [61]. The increase in G″ is explained by the fact that more deformation energy is lost due to internal friction during shearing. The breakdown of the micellar structure starts with the disassembly of macromolecules which have internal viscous friction, thus converting the deformation energy into friction. The decrease in the mechanical strength of the gel by the polymer micelles can be considered an advantage of the material in cases when the gel is applied to wounds by smearing. Thus, the wound will be covered easily with less effort. The rheological measurements confirmed that the formulation containing 8% HPC, 2% F127 and 1% OEO possesses favorable characteristics for further evaluation of topical delivery of OEO.

### 2.4. Occlusion Test

**Figure 4:**
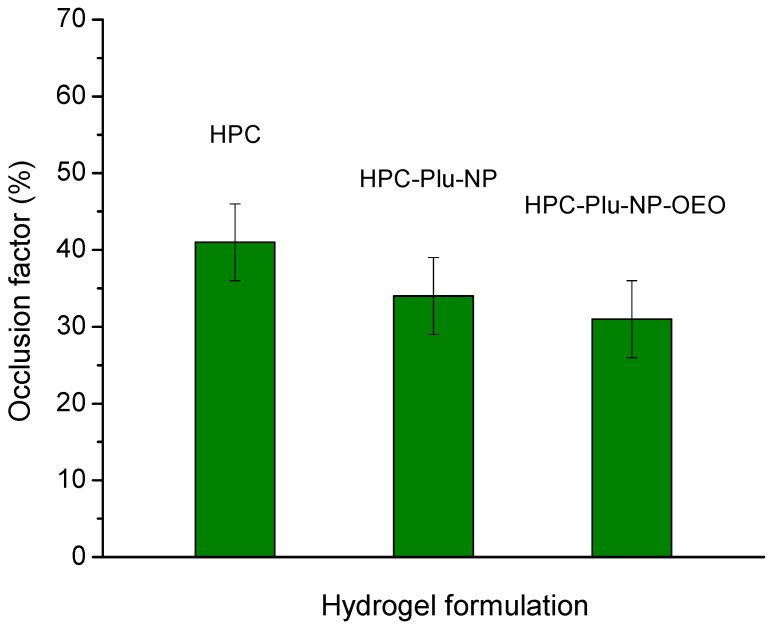
Occlusion factor for the empty HPC-hydrogel (8%), Pluronic F127 nanocomposite hydrogel (8% HPC-NP-Plu) and OEO/F127 nanocomposite hydrogel (8% HPC-NP-Plu-OEO).

The occlusive properties of formulations for local skin treatment are related to their ability to form a continuous and dense film on the skin surface which has a direct impact on the normal transepidermal water loss. An occlusive factor of 0 means no occlusion, while an occlusive factor of 100 indicates maximum occlusive effect. The skin occlusion may improve stratum corneum hydration and enhance percutaneous absorption of the active ingredient, as the drug diffusion rate is higher through hydrated skin [25,62,63]. Our in vitro test showed that the pure HPC hydrogel and nanocomposite hydrogels, containing blank and OEO-loaded F127 micelles, have relatively close occlusive factors, in a range from 31 to 41 (Figure 4). Similar values of the occlusion factor of plain and nanoparticle-containing Pluronic-based gels were recently reported by Slavkova et al. [64]. Such results indicate that the developed hydrogel formulations can ensure a favorable combination of breathability and hydration ability, which are considered important parameters for effective wound healing.

### 2.5. In Vitro Release of Oregano Essential Oil

**Figure 5:**
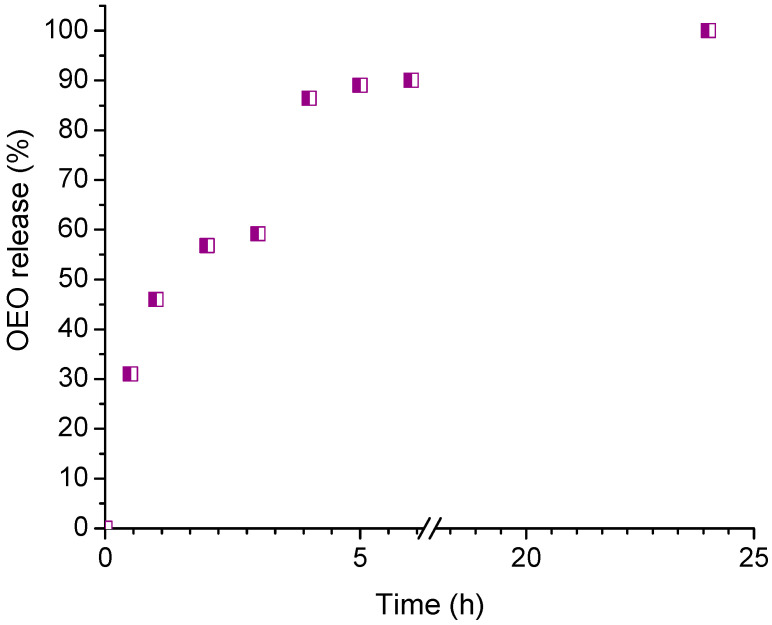
In vitro release of oregano essential oil from 8% HPC-Plu-NP-OEO nanocomposite hydrogels in a buffer medium (pH 7).

The physiological pH of healthy skin which has a thin acidic mantle is between 4 and 6. Melanocytes in the epidermis that are located below this protective layer have a pH of about 7.4, while the pH of melanoma cells is approximately 7 [65,66]. Therefore, the in vitro release test was carried out in a buffer medium with pH 7. The release profile of OEO from 8% HPC nanocomposite hydrogel is shown in Figure 5. We found that the polymer nanocomposite gel carrier provided a sustained release of OEO over a period of 24 h. Recently, Avram et al. reported a hydrogel delivery system of OEO based on two Pluronics (binary mixture of F127 and L31), which released approximately 35% of the oil in 7 h [67]. It seems that the HPC hydrogel matrix, which is much more hydrated than a Pluronic gel, provides relatively rapid diffusion of OEO molecules, as 96% of the encapsulated OEO was released by the gel in 8 h.

### 2.6. Cytotoxicity and Antiproliferative Activity

**Figure 6:**
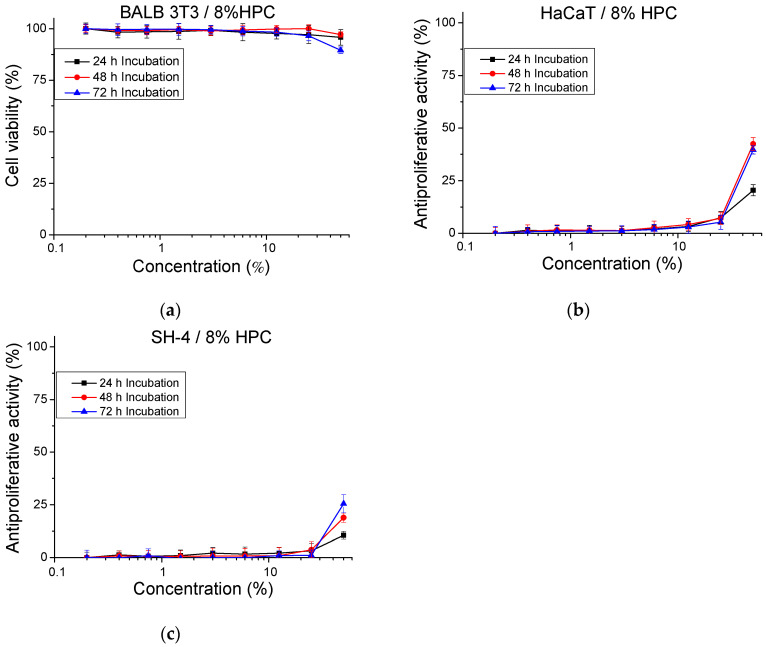
In vitro assessment of the cytotoxicity (**a**) and antiproliferative effect of 8% HPC gel on HaCaT (**b**) and SH-4 (**c**) cells. Cells were exposed to the sample extract (DMEM incubated with 8% HPC gel for 24 h, 48 h and 72 h) in different concentrations (from 0.2% to 50%). *n* = 6.

Preliminary experiments performed with normal cells were aimed at confirming that the polymeric carriers used in our study are devoid of toxicity. Thus, 5 mL of cell culture medium (DMEM) were added to 1 mL of 8% HPC gel. The gel/medium system was incubated at 37 °C, 5% CO_2_ for 72 h. Samples were taken for in vitro analysis at each 24 h increment (24, 48 and 72 h) of the incubation time. Nine different concentrations of the samples, obtained by diluting the hydrogel sample with the addition of fresh culture medium (from 0.2% to 50%), were investigated. The cytotoxicity of the samples was tested on mouse embryonic fibroblasts (BALB 3T3) and the results are presented as a percentage of the negative control (Figure 6a). In the culture medium incubated with 8% HPC for 24 h and 48 h, no cytotoxic effect was observed even at the highest concentrations tested. In the medium incubated with 8% HPC for 72 h, a marginal cytotoxic effect (10.45%) was detected only at the highest tested concentration (50%). Noteworthy is that, at the lower concentrations, the measured toxicity did not differ from the negative control (*p* > 0.05).

**Figure 7:**
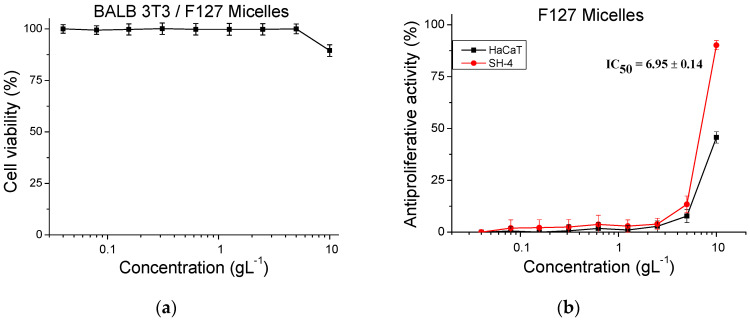
In vitro assessment of the cytotoxicity (**a**) and antiproliferative activity of blank F127 micelles on HaCaT and SH-4 cells (**b**) at different concentrations. *n* = 6.

In addition, tests for determining the antiproliferative activity of pure 8% HPC hydrogel on a normal human keratinocyte cell line (HaCaT) and melanoma tumor cell line (SH-4) (Figure 6b,c) were carried out. In fact, antiproliferative activity was observed only at the highest tested concentration (50%). In this case, the longer the incubation time, the higher the inhibition of the cell proliferation. In vitro tests were also performed to assess the cytotoxicity and antiproliferative activity of F127 micelles (Figure 7). The micelles were tested in a wide range of concentrations from 0.04 to 10 gL^−1^. The experiments confirmed that F127 micelles are not cytotoxic at the studied concentrations, and even at the highest tested concentration of 10 gL^−1^, the calculated cytotoxicity was 10%. At this concentration, we also observed an antiproliferative effect on both normal keratinocytes (HaCaT) (45%) and tumor SH-4 cells (90%) (Figure 7b).

**Figure 8:**
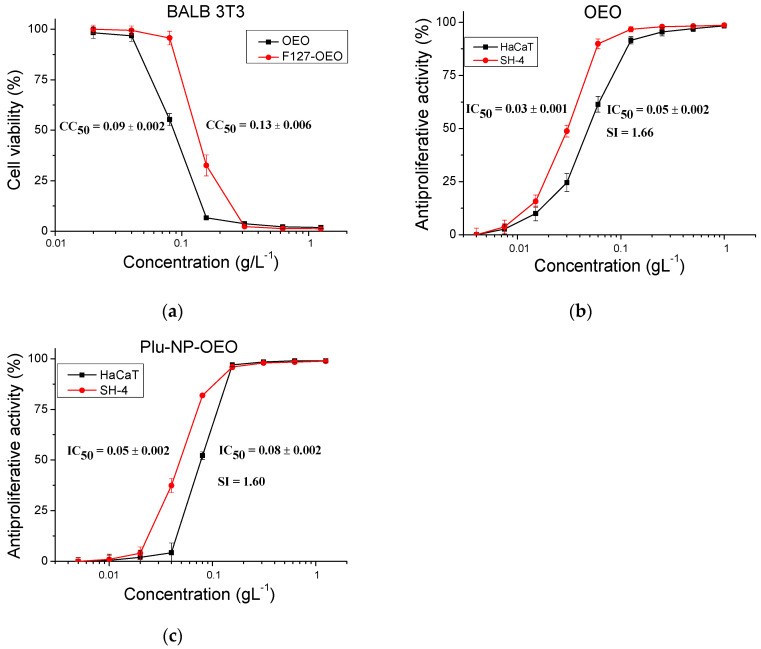
In vitro assessment of the cytotoxicity (**a**) and antiproliferative activity of pure OEO (**b**) and OEO-loaded F127 micelles (**c**) on HaCaT and SH-4 cells, at different sample concentrations. *n* = 6.

Next, the cytotoxicity and antiproliferative activity of pure oregano essential oil and OEO-loaded F127 micelles were studied in a comparative aspect. This provided us with information about the change in the activity of oregano oil that occurs as a result of micelle formation. The cytotoxicity test (Figure 8a) revealed a notable reduction in the cytotoxicity of OEO when it was added to the cell culture in its micellar form (F127–OEO). The calculated average value of CC_50_ for pure and formulated OEO was 0.09 and 0.13 gL^−1^, respectively. The estimated cytotoxicity reduction in OEO when loaded into F127 micelles was 44% (((0.13 − 0.09)/0.09) × 100 = 44%).

**Figure 9:**
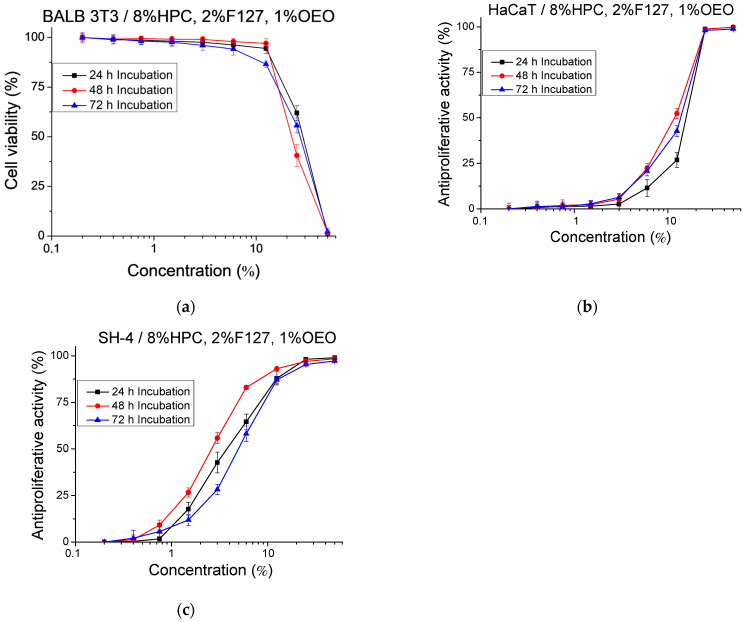
In vitro assessment of cytotoxicity (**a**) and antiproliferative activity of HPC hydrogel containing OEO-loaded F127 micelles (8% HPC, 2% F127, 1% OEO) on HaCaT (**b**) and SH-4 cells (**c**). Cells were exposed to the sample extract (DMEM incubated with 8% HPC, 2% F127, 1% OEO for 24 h, 48 h and 72 h) in different concentrations (from 0.2% to 50%). *n* = 6.

Similarly, the antiproliferative activity of the micellar formulation of OEO on normal and tumor cell lines (Figure 8b,c) was less pronounced compared to the pure oil. We also found that the selectivity of pure OEO (SI = 1.67) was higher than the selectivity of the micellar formulation (SI = 1.60). Finally, we investigated the cytotoxicity and antiproliferative activity of HPC hydrogel containing OEO-loaded F127 micelles (8% HPC, 2% F127, 1% OEO) at the conditions reported for pure HPC hydrogel. At the highest tested concentration, 100% toxicity was observed in the three samples, taken at 24 h, 48 h and 72 h of incubation, respectively (Figure 9a). On the other hand, no cytotoxicity was observed in any of the tested samples when the concentration was ≤12.5%.

The results of the antiproliferative activity test (Figure 9b,c) showed that the nanogel system had the strongest effect at 48 h of incubation. The weaker effect at 24 h of incubation is probably due to the delayed release of the essential oil from the polymer carrier. In addition, we observed selectivity towards tumor cells (SH-4) of the three samples taken at 24 h, 48 h and 72 h of incubation (Table 4). The highest selective index (SI) was found for the sample taken at 48 h of incubation (SI = 4.5).

## 3. Conclusions

A water-soluble form of *Origanum vulgare* ssp. *hirtum* essential oil was successfully prepared by encapsulating the oil in Pluronic F127 micelles. Stable aqueous colloids were obtained at polymer/oil mass ratios ≥ 1.7. The micellar OEO was then easily embedded into a physical HPC hydrogel, yielding nanocomposite gels with a smooth texture. Although the presence of OEO-loaded F127 micelles decreased to some extent the mechanical strength of HPC hydrogel, a formulation based on 8% HPC, 2% F127 and 1% OEO exhibited favorable mechanical and occlusive characteristics for topical delivery of OEO. In vitro experiments with normal and skin cancer cells showed that the polymeric carriers (HPC and Pluronic F127) are devoid of cytotoxicity, while the pure essential oil possesses high toxicity and has a notable antiproliferative effect. Encapsulation of OEO in micelles and gel carriers reduced the cytotoxicity of the oil but maintained its therapeutic potential. Moreover, the nanogel formulation showed pronounced selectivity against human melanoma cells, making it an excellent candidate for application as anticancer agent.

## 4. Materials and Methods

### 4.1. Materials

Hydroxypropyl cellulose (Klucel™ MF Pharm, Mw = 1,000,000 Da), poly(ethylene glycol)-block-poly(propylene glycol)-block-poly(ethylene glycol) (Pluronic F127, Mn =12 600 g/mol), ethanol (99.5%) and phosphate buffer pH = 7 were purchased from Sigma-Aldrich via FOT, Sofia, Bulgaria, and used as received.

### 4.2. Plant Material and Essential Oil Distillation

Vegetatively propagated plants of line 149/4/iv3 of *O. vulgare* ssp. *hirtum*, grown in the experimental field of the AgroBioInstitute in nearby Kostinbrod, Bulgaria, were used in the study. Line 149/4/iv3 was selected in 2019 from a wild population in the Rhodope Mountains, Bulgaria [68]. The taxonomic affiliation of the plant was determined by DNA barcoding following sequencing of regions from the chloroplast genome including the rubisco large subunit (rbcL) and maturase K (matK) genes and the intergenic spacer trnH-psbA, as well as by visual assessment of the plant appearance. OEO was produced by hydrodistillation of inflorescences of the flowering plants using a Clevenger apparatus. The distillation of OEO was carried out using 20 g of oregano inflorescences in 200 mL water for a period of 2 h. Ten batches of distilled OEO were combined to produce the final OEO sample used in the experiments.

### 4.3. GC–MS/FID Analysis of Oregano Essential Oil

A total of 20 microliters of OEO were diluted with 0.38 mL hexane and the obtained diluted OEO was used for GC–MS/FID analysis. The analysis was performed on an Agilent 8890 GC system equipped with an Agilent 5977B mass spectrometer with a FID detector (Agilent Technologies, Santa Clara, CA, USA). The compounds were separated on an Agilent HP-INNOWax column (30 m × 0.25 mm, 0.25 μm) utilizing PEG as a stationary phase with helium 5.0 (purity 99.999 vol.%) as a carrier gas at a constant flow of 0.8 mL/min. One microliter of essential oil was injected using a split of 1:100 and the following acquisition parameters: injector temperature 250 °C. Oven program: initial temperature 65 °C, then 2 °C/min to 170 °C, hold for 0 min, then 60 °C/min to 240 °C, hold for 15 min, run time 68.667 min. The 5977B mass-selective detector was operated at a transfer line temperature of 250 °C, electron impact ionization voltage of 70 eV and quadrupole temperature of 150 °C. The FID detector was operated at 300 °C. Normal alkanes C10–C40 (Sigma-Aldrich via FOT, Sofia, Bulgaria) were used for RI calculation using AMDIS ver. 2.71 (National Institute of Standards and Technology (NIST), USA). Compound identification was carried out based on comparison of their mass spectrum and RI data with the NIST 2008 Mass spectral library (National Institute of Standards and Technology (NIST), USA) and literature data. Relative quantification of each compound expressed as percentage of the total chromatogram area was performed based on data from the FID detector.

The composition of the oil after loading into the polymer nanocarriers was determined by the same procedure.

### 4.4. Preparation of Blank and OEO-Loaded Micelles

Pluronic F127 and *Origanum vulgare* ssp. *hirtum* essential oil were dissolved in ethanol and the solution was added dropwise to 10 mL of water. After stirring at room temperature on a magnetic stirrer for 30 min, the ethanol was removed under vacuum. Six samples were prepared at a polymer concentration of 1% *w*/*w* (10 gL^−1^) and polymer/OEO mass ratios of 2, 1.8, 1.7, 1.5, 1.2 and 1. Next, 1 sample was obtained with an F127 concentration of 2% *w*/*w* (20 gL^−1^) and a polymer/OEO mass ratio of 2. Aqueous solutions of blank F127 micelles were prepared by the same procedure but without adding OEO.

### 4.5. Preparation of Pure HPC Hydrogel and Nanocomposite HPC Hydrogels with OEO-Loaded Micelles

Nanocomposite hydrogels (4 and 8% *w*/*w*) were prepared by dissolving HPC in 1 mL of the micellar solutions (with and without OEO) at a mass ratio of 4 for the two polymers (HPC/Pluronic 127). The fabricated samples were stirred for 30 min at room temperature and then kept overnight at room temperature before measurements.

The same procedure was used for the preparation pure HPC solutions/gels (1–10% *w*/*w*) except that water was used instead of a micellar solution.

### 4.6. Analysis

The hydrodynamic diameter of micellar nanocarriers was investigated by dynamic light scattering (DLS) measurements using a Zetasizer NanoBrook 90Plus PALS instrument (Brookhaven Instruments, Holtsville, NY, USA) equipped with a 35 mW red diode laser (λ = 640 nm) at 25 °C and a scattering angle of 90°. The ζ potential values were determined by the phase analysis light scattering (PALS) method, conducted on the same instrument. Dynamic rheological measurements were conducted with a HAAKE MARS 60 rheometer (Thermo Fisher Scientific, Waltham, MA, USA). Rheological characterization of the hydrogels was conducted with a HAAKE MARS 60 rheometer (Thermo Fisher Scientific, Waltham, MA, USA). The storage (G′) and loss (G′′) moduli were determined at constant deformation (γ = 0.01) in the 0.1–10 Hz frequency range. Dynamic oscillatory amplitude tests were carried out by varying the shear strain from 0.001 to 10 at a frequency of 1 Hz. All rheological experiments were carried out at 32 °C.

### 4.7. Occlusion Factor Determination

The occlusion factor (F) of blank HPC hydrogels (8%), nanocomposite hydrogels (8% HPC-Plu-NP) and OEO-loaded nanocomposite hydrogels (8% HPC-Plu-NP-OEO) was determined in vitro according to a procedure described elsewhere [65]. A 50 mL beaker was filled halfway with distilled water (25 mL), covered with filter paper (Whatman cellulose filter (0.45 µm)) and sealed with Teflon tape. An equal amount of each gel formulation was applied as an even thin film on the filter paper surface. A beaker containing 25 mL of distilled water but without the gel sample was used as reference. After 48 h, the beakers were weighed, and water loss (evaporated through the filter) was calculated. The occlusion factor was calculated using the follow equation:

$$F\left(\%\right)=\frac{A-B}{A}\times 100$$

where A is water loss of the reference sample and B is water loss of the gel [64].

### 4.8. In Vitro Release of Oregano Essential Oil

In vitro release of OEO from the nanogel formulation was studied in a buffer medium (pH = 7) at 32 °C. For this purpose, 5 mL of the release medium was added to 1 mL of the OEO-loaded hydrogel (8% HPC-Plu-NP-OEO), containing 100 mg of OEO, in flat-bottom glass bottles. The bottles were transferred into a thermostatic bath at 32 °C. At defined time intervals, aliquot samples (20 mL) were extracted and replaced with fresh medium. The released OEO amount was evaluated using the GC method by monitoring the concentration of carvacrol in the sample.

### 4.9. In Vitro Cytotoxicity and Antiproliferative Activity Studies

#### 4.9.1. Cell Cultures

The mouse embryonic fibroblasts BALB/3T3 clone A31 (ATCC^®^ CCL-163^TM^) and human melanoma SH-4 (ATCC^®^ CRL-7724^TM^) cell lines were obtained from American Type Cultures Collection (ATCC, Manassas, VA, USA). The human keratinocytes HaCaT (CLS, cat. № 300493) were obtained from CLS Cell Lines Service GmbH (CLS, Eppelheim, Germany). Dulbecco’s modified Eagle’s medium (DMEM), fetal bovine serum (FBS), antibiotics (penicillin and streptomycin), trypsin-EDTA solution, Neutral Red (NR) and MTT-dye were purchased from Sigma-Aldrich, Schnelldorf, Germany. The disposable consumables were supplied by Orange Scientific, Braine-l’Alleud, Belgium. Cells were cultured in DMEM 4.5 g/L glucose, supplemented with 10% (*v*/*v*) FBS, 100 U/mL penicillin and 0.1 mg/mL streptomycin in an incubator at 37 °C, 5% CO_2_ and 95% humidity. The cells were grown using 75 cm^2^ plastic flasks.

#### 4.9.2. Cytotoxicity

The cytotoxicity test was performed as described by Borenfreund [69] and the latest modification of the BALB 3T3 Neutral Red Uptake Cytotoxicity Assay [70]. BALB 3T3 cells were plated at a density of 1 × 10^4^ cells in 100 μL culture medium in each well of 96-well microplates and allowed to adhere for 24 h before treatment with test compounds. A wide concentration range was applied, and the cells were incubated for an additional 24 h. After treatment with NR-Dye medium, washing and application of the NR Desorb solution (1% glacial acetic acid, 50% ethanol and 49% dH_2_O). The absorption was measured on a TECAN microplate reader at a wavelength of 540 nm. Cell viability is presented as a percentage of the negative control.

#### 4.9.3. Antiproliferative Activity

The antiproliferative activity test was performed on cell cultures using the MTT-dye reduction assay [71]. Cell lines HaCaT and SH-4 were used in the experiments. Cells were plated at a density of 1 × 10^3^ cells/100 µL in each well of 96-well microplates and allowed to adhere for 24 h. The cells were then incubated for 72 h with the test compounds dissolved in growth medium (DMEM, 10% FBS). The MTT-formazan absorption was registered using a microplate reader at λ = 540 nm. Antiproliferative activity is expressed as IC_50_ values (concentrations required for 50% inhibition of cell growth), calculated using non-linear regression analysis by GraphPad Prizm 8 Software (San Diego, CA, USA).

#### 4.9.4. Statistical Analysis

The statistical analysis included application of one-way ANOVA followed by Bonferroni’s post hoc test. *p* < 0.05 was accepted as the lowest level of statistical significance. All results are presented as mean ± SD.

## Figures and Tables

**Table 1:** Calculation of the concentration of the main compounds of OEO by GC–MS, before and after loading into polymeric micelles.

Compound	Rt (min)	Concentration before Loading (% of Total Peak Areas)	Concentration after Loading (% of Total Peak Areas)
Carvacrol	58.66	90.31	90.28
Thymol	58.18	1.62	1.62

**Table 2:** Hydrodynamic diameter, ζ potential and transmittance of OEO-loaded polymeric nanocarriers at different polymer/OEO mass ratios and constant concentration of Pluronic F127 = 1% *w*/*w*.

Sample	Mass Ratio F127/OEO	D_h_ (nm)	ζ-Potential (mV)	Transmittance (%)
1	2	34 ± 2	−3.16 ± 0.55	85.35
2	1.8	36 ± 2	−2.46 ± 0.24	56.52
3	1.7	67 ± 3	−0.52 ± 0.14	1.76
4	1.5	-	-	0.26
5	1.2	-	-	0.03
6	1	-	-	0.01
F127	-	25 ± 1	−6.91 ± 1.32	-

**Table 3:** Elastic (G′) and loss (G″) moduli and complex dynamic viscosity (η*) of HPC solutions/gels at different concentrations of polymer (from 1% to 10% *w*/*w*).

	HPC Concentration									
	1%	2%	3%	4%	5%	6%	7%	8%	9%	10%
G′ (Pa)	1.3	18.7	53.3	201.6	773.8	897.4	1368.0	2341.2	2426.9	2817.4
G″ (Pa)	6.1	32.9	61.6	176.7	494.3	502.7	757.6	1140.3	1108.6	1133.1
η* (Pa·s)	0.9	6.0	12.9	42.6	146.1	163.7	248.9	414.3	424.4	483.2

**Table 4:** Mean IC_50_ ± SD and selective index (SI).

HPC/F127/OEO	Mean IC_50_ ± SD (%)			SI *
	Cytotoxicity	Antiproliferative Activity		
	BALB 3T3	HaCaT	SH-4	
24 h	28.63 ± 0.96	15.61 ± 0.45	3.78 ± 0.62	4.13
48 h	22.36 ± 1.38	11.84 ± 0.73	2.63 ± 0.16	4.50
72 h	26.83 ± 1.13	13.70 ± 0.46	5.02 ± 0.36	2.73

* SI = IC_50_ (normal cell line HaCaT)/IC_50_ (tumor cell line SH-4).

## Data Availability

The raw data supporting the conclusions of this article will be made available by the authors on request.

## Supplementary Materials

The following supporting information can be downloaded at https://www.mdpi.com/article/10.3390/gels10100627/s1, Table S1. Chemical composition of *Origanum vulgare* ssp. *hirtum* essential oil determined by GC–MS analysis; Figure S1. Plot of the transmittance (a) and digital image (b) of OEO-loaded polymeric micelles (Plu-NP-OEO) at different F127/OEO mass ratio; Figure S2. Hydrodynamic diameter of empty (Plu-NP) and OEO-loaded polymeric micelles (Plu-NP-OEO) at different polymer/OEO mass ratio—2, 1.8 and 1.7 and constant concentration of Pluronic F127 (1% *w*/*v*).
