# Supplementary material for: Hydroxypropyl Cellulose Hydrogel Containing Origanum vulgare ssp. hirtum Essential-Oil-Loaded Polymeric Micelles for Enhanced Treatment of Melanoma
Source: Gels. 2024 Sep 29;10(10):627. doi: 10.3390/gels10100627 (PMC11508108; doi:10.3390/gels10100627)
Supplement: Supplementary file 1 [file gels-10-00627-s001.zip › gels-3210779-supplementary.pdf]

Supplementary Materials:

Hydroxypropyl cellulose hydrogel containing *Origanum vulgare ssp. hirtum* essential oil-loaded polymeric micelles for enhanced treatment of melanoma

Katya Kamenova[1], Ivan Iliev[2], Anna Prancheva[1], Pencho Tuleshkov[1], Krasimir Rusanov[3], Ivan Atanassov[2], Petar D. Petrov[1,*]

Table S1. Chemical composition of Origanum vulgare ssp. hirtum essential oil determined by GC–MS analysis.

	Compound	RT	RI	%
1	α-Pinene	8.472	1006.3	0.034
2	α-Thujene	8.598	1009.7	0.067
3	β-Myrcene	13.8764	1152.2	0.1
4	α-Terpinene	14.6221	1172.3	0.044
5	γ-Terpinene	18.0297	1242.5	0.312
6	3-Octanone	18.5001	1250.9	0.098
7	p-Cymene	19.4545	1267.9	0.473
8	1-Octen-3-ol	29.9472	1451.9	0.58
9	cis-Sabinene hydrate	30.7156	1464.8	1.069
10	Linalool	35.8088	1550.3	0.372
11	Caryophyllene	38.4927	1595.4	0.874
12	Terpinen-4-ol	38.92	1602.7	0.361
13	Carvacrol methyl ether	39.236	1608.4	0.417
14	trans-Dihydrocarvone	40.3269	1627.8	0.165
15	Humulene	42.6583	1669.3	0.187
16	α-Terpineol	44.3779	1700	0.133
17	endo-Borneol	44.5371	1702.8	0.976
18	β-Bisabolene	46.0123	1729.1	0.379
19	Carvone	46.3356	1734.9	0.191
20	α-Farnesene	47.3274	1752.5	0.109
21	Caryophyllene oxide	55.2654	1992.8	0.18
22	Spathulenol	57.4172	2140.6	0.075
23	Thymol	58.1816	2195.6	1.618
24	Carvacrol	58.6649	2226.8	90.306
	Total identified			99.12

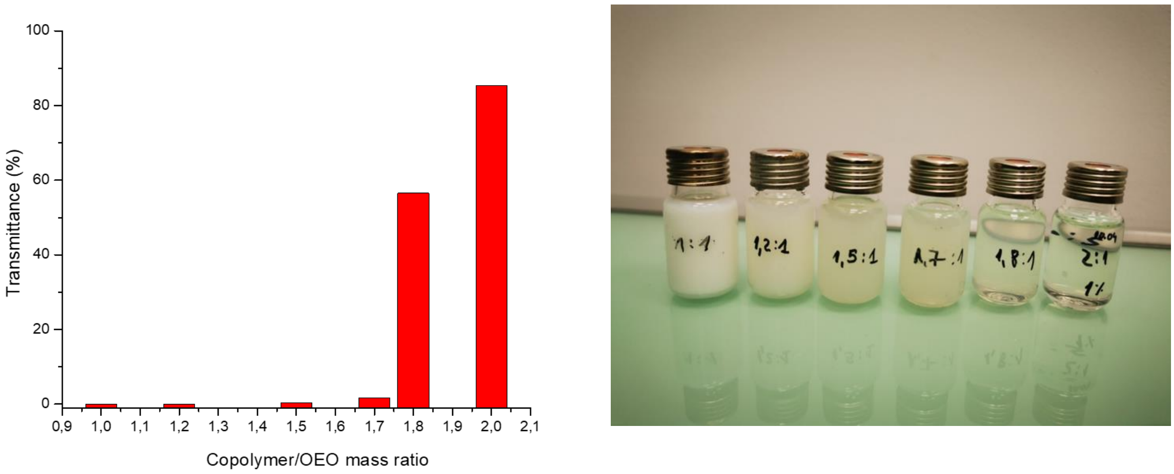

(**a**) (**b**)

Figure S1. Plot of the transmittance (a) and digital image (b) of OEO-loaded polymeric micelles (Plu-NP-OEO) at different F127/OEO mass ratio.

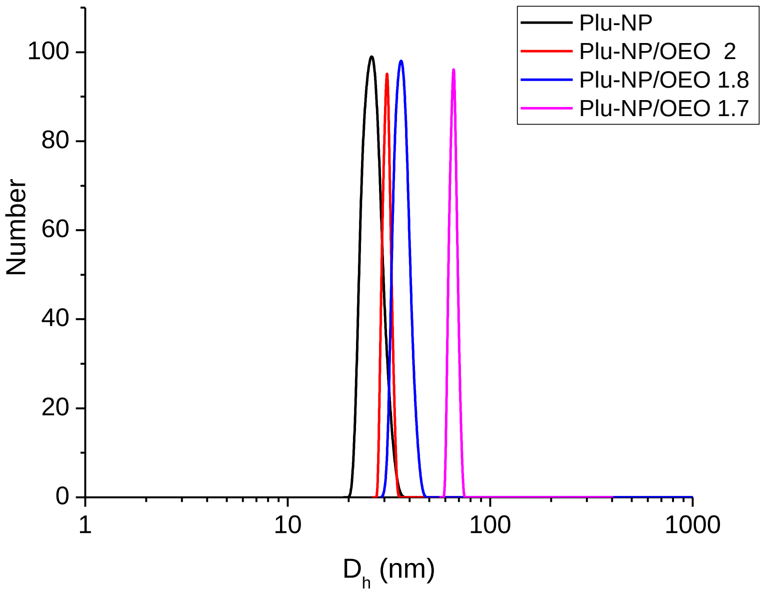

Figure S2. Hydrodynamic diameter of empty (Plu-NP) and OEO-loaded polymeric micelles (Plu-NP-OEO) at different polymer/OEO mass ratio - 2, 1.8 and 1.7 and constant concentration of Pluronic F127 (1% w/v).
